# Bis[4-(dimethyl­amino)pyridinium] tetra­bromidocuprate(II)

**DOI:** 10.1107/S1600536809028128

**Published:** 2009-07-22

**Authors:** Kong Mun Lo, Seik Weng Ng

**Affiliations:** a Department of Chemistry, University of Malaya, 50603 Kuala Lumpur, Malaysia

## Abstract

The metal atom in the anion of the title salt, (C_7_H_11_N_2_)_2_[CuBr_4_], shows a distorted tetra­hedral coordination. The primary contacts between the ions are of the N—H⋯Br type.

## Related literature

For other pyridinium tetra­bromidocuprates, see: Coffey *et al.* (1996 ▶); Haddad & Al-Far (2008 ▶); Luque *et al.* (2001 ▶); Willet *et al.* (2000 ▶, 2003 ▶).
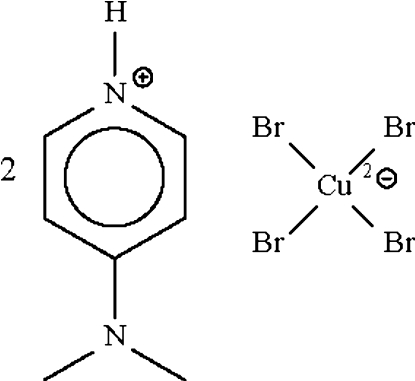


## Experimental

### 

#### Crystal data

(C_7_H_11_N_2_)_2_[CuBr_4_]
*M* *_r_* = 629.54
Triclinic, 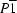

*a* = 8.1768 (2) Å
*b* = 9.2406 (3) Å
*c* = 14.3686 (4) Å
α = 93.689 (2)°
β = 94.814 (2)°
γ = 105.073 (2)°
*V* = 1040.42 (5) Å^3^
*Z* = 2
Mo *K*α radiation
μ = 8.73 mm^−1^
*T* = 233 K
0.35 × 0.30 × 0.10 mm

#### Data collection

Bruker SMART APEX diffractometer
Absorption correction: multi-scan (*SADABS*; Sheldrick, 1996 ▶) *T*_min_ = 0.321, *T*_max_ = 0.746 (expected range = 0.180–0.418)
7224 measured reflections
4595 independent reflections
3168 reflections with *I* > 2σ(*I*)
*R*_int_ = 0.042

#### Refinement

*R*[*F* ^2^ > 2σ(*F* ^2^)] = 0.043
*wR*(*F* ^2^) = 0.123
*S* = 1.00
4595 reflections
213 parameters
H-atom parameters constrained
Δρ_max_ = 0.84 e Å^−3^
Δρ_min_ = −0.82 e Å^−3^

### 

Data collection: *APEX2* (Bruker, 2008 ▶); cell refinement: *SAINT* (Bruker, 2008 ▶); data reduction: *SAINT*; program(s) used to solve structure: *SHELXS97* (Sheldrick, 2008 ▶); program(s) used to refine structure: *SHELXL97* (Sheldrick, 2008 ▶); molecular graphics: *X-SEED* (Barbour, 2001 ▶); software used to prepare material for publication: *publCIF* (Westrip, 2009 ▶).

## Supplementary Material

Crystal structure: contains datablocks global, I. DOI: 10.1107/S1600536809028128/tk2503sup1.cif

Structure factors: contains datablocks I. DOI: 10.1107/S1600536809028128/tk2503Isup2.hkl

Additional supplementary materials:  crystallographic information; 3D view; checkCIF report

## Figures and Tables

**Table 1:** Hydrogen-bond geometry (Å, °)

*D*—H⋯*A*	*D*—H	H⋯*A*	*D*⋯*A*	*D*—H⋯*A*
N1—H1⋯Br1	0.88	2.54	3.380 (7)	162
N3—H3⋯Br2	0.88	2.65	3.449 (6)	152

**Fig. 1.**
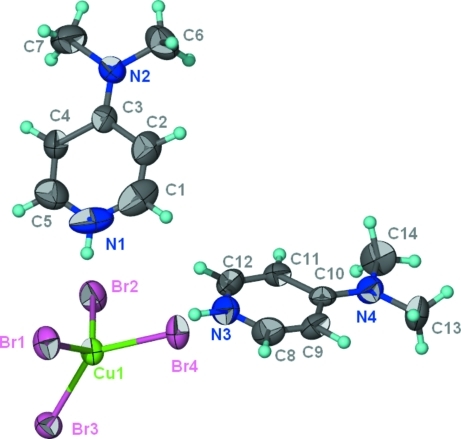
Thermal ellipsoid plot (Barbour, 2001) of 2[C7H11N2][CuBr4] at the 50% probability level. Hydrogen atoms are drawn as spheres of arbitrary radius.

## supplementary crystallographic information

### Experimental

Copper sulfate pentahydrate (2.1 g, 8.3 mmol) dissolved in water (5 ml) was mixed with  4-dimethylaminopyridine hydrobromide perbromide (3 g, 8.3 mmol) dissolved in ethanol (10 ml).  The mixture was heated for 30 min.  The filtered green solution when allowed to evaporate yielded black crystals.

### Refinement

Hydrogen atoms were placed at calculated positions (C–H 0.94–0.97 Å; N–H 0.88 Å) and were treated as riding on their parent atoms, with *U*(H) set to 1.2–1.5*U*_eq_(C, N).

### Crystal data

**Table:**

(C_7_H_11_N_2_)_2_[CuBr_4_]	*Z* = 2
*M**_r_* = 629.54	*F*(000) = 606
Triclinic, *P*1	*D*_x_ = 2.010 Mg m^−^^3^
Hall symbol: -P 1	Mo *K*α radiation, λ = 0.71073 Å
*a* = 8.1768 (2) Å	Cell parameters from 2442 reflections
*b* = 9.2406 (3) Å	θ = 2.3–27.5°
*c* = 14.3686 (4) Å	µ = 8.73 mm^−^^1^
α = 93.689 (2)°	*T* = 233 K
β = 94.814 (2)°	Block, black
γ = 105.073 (2)°	0.35 × 0.30 × 0.10 mm
*V* = 1040.42 (5) Å^3^	

### Data collection

**Table:**

Bruker SMART APEX diffractometer	4595 independent reflections
Radiation source: fine-focus sealed tube	3168 reflections with *I* > 2σ(*I*)
graphite	*R*_int_ = 0.042
ω scans	θ_max_ = 27.5°, θ_min_ = 1.4°
Absorption correction: multi-scan (*SADABS*; Sheldrick, 1996)	*h* = −10→10
*T*_min_ = 0.321, *T*_max_ = 0.746	*k* = −10→12
7224 measured reflections	*l* = −18→18

### Refinement

**Table:**

Refinement on *F*^2^	Secondary atom site location: difference Fourier map
Least-squares matrix: full	Hydrogen site location: inferred from neighbouring sites
*R*[*F*^2^ > 2σ(*F*^2^)] = 0.043	H-atom parameters constrained
*wR*(*F*^2^) = 0.123	*w* = 1/[σ^2^(*F*_o_^2^) + (0.0566*P*)^2^] where *P* = (*F*_o_^2^ + 2*F*_c_^2^)/3
*S* = 1.00	(Δ/σ)_max_ = 0.001
4595 reflections	Δρ_max_ = 0.84 e Å^−^^3^
213 parameters	Δρ_min_ = −0.82 e Å^−^^3^
0 restraints	Extinction correction: *SHELXL97* (Sheldrick, 2008), Fc^*^=kFc[1+0.001xFc^2^λ^3^/sin(2θ)]^-1/4^
Primary atom site location: structure-invariant direct methods	Extinction coefficient: 0.0078 (8)

### Fractional atomic coordinates and isotropic or equivalent isotropic displacement parameters (Å^2^)

**Table:**

	*x*	*y*	*z*	*U*_iso_*/*U*_eq_	
Br1	0.46757 (8)	0.23274 (7)	0.31534 (5)	0.04383 (19)	
Br2	0.44673 (8)	0.69937 (7)	0.29470 (5)	0.0458 (2)	
Br3	0.11950 (8)	0.35808 (7)	0.20247 (4)	0.03895 (18)	
Br4	0.65667 (8)	0.48740 (7)	0.15029 (4)	0.04107 (19)	
Cu1	0.41731 (9)	0.44301 (7)	0.23676 (5)	0.0323 (2)	
N1	0.8043 (9)	0.5220 (8)	0.4011 (5)	0.067 (2)	
H1	0.7335	0.4434	0.3685	0.081*	
N2	1.1304 (7)	0.8919 (6)	0.5502 (4)	0.0466 (13)	
N3	0.6251 (7)	0.8455 (6)	0.1004 (4)	0.0478 (13)	
H3	0.5794	0.7776	0.1376	0.057*	
N4	0.8436 (7)	1.1604 (6)	−0.0731 (3)	0.0415 (12)	
C1	0.9472 (12)	0.5930 (9)	0.3673 (5)	0.063 (2)	
H1A	0.9709	0.5576	0.3085	0.075*	
C2	1.0582 (9)	0.7127 (9)	0.4141 (5)	0.0521 (18)	
H2	1.1589	0.7596	0.3887	0.062*	
C3	1.0231 (7)	0.7695 (7)	0.5032 (4)	0.0343 (13)	
C4	0.8711 (8)	0.6903 (8)	0.5360 (5)	0.0465 (16)	
H4	0.8409	0.7213	0.5942	0.056*	
C5	0.7692 (9)	0.5702 (9)	0.4836 (5)	0.064 (2)	
H5	0.6683	0.5179	0.5067	0.077*	
C6	1.2928 (10)	0.9673 (9)	0.5180 (6)	0.067 (2)	
H6A	1.2723	1.0101	0.4597	0.100*	
H6B	1.3585	0.8951	0.5080	0.100*	
H6C	1.3556	1.0469	0.5650	0.100*	
C7	1.0967 (10)	0.9465 (9)	0.6426 (5)	0.062 (2)	
H7A	0.9814	0.9571	0.6393	0.093*	
H7B	1.1767	1.0433	0.6623	0.093*	
H7C	1.1095	0.8752	0.6875	0.093*	
C8	0.6803 (9)	0.8036 (7)	0.0201 (5)	0.0472 (17)	
H8	0.6688	0.7008	0.0045	0.057*	
C9	0.7497 (8)	0.9012 (7)	−0.0374 (4)	0.0409 (15)	
H9	0.7858	0.8671	−0.0931	0.049*	
C10	0.7710 (7)	1.0591 (6)	−0.0161 (4)	0.0328 (13)	
C11	0.7118 (7)	1.1009 (7)	0.0682 (4)	0.0374 (14)	
H11	0.7217	1.2026	0.0863	0.045*	
C12	0.6401 (8)	0.9918 (8)	0.1234 (4)	0.0462 (17)	
H12	0.5999	1.0200	0.1793	0.055*	
C13	0.9027 (9)	1.1174 (9)	−0.1615 (5)	0.0558 (18)	
H13A	0.9415	1.0274	−0.1553	0.084*	
H13B	0.9960	1.1986	−0.1764	0.084*	
H13C	0.8099	1.0977	−0.2112	0.084*	
C14	0.8708 (10)	1.3216 (6)	−0.0511 (5)	0.059 (2)	
H14A	0.9513	1.3551	0.0044	0.088*	
H14B	0.7635	1.3424	−0.0398	0.088*	
H14C	0.9158	1.3747	−0.1035	0.088*	

### Atomic displacement parameters (Å^2^)

**Table:**

	*U*^11^	*U*^22^	*U*^33^	*U*^12^	*U*^13^	*U*^23^
Br1	0.0494 (4)	0.0387 (4)	0.0482 (4)	0.0151 (3)	0.0101 (3)	0.0186 (3)
Br2	0.0496 (4)	0.0355 (3)	0.0513 (4)	0.0073 (3)	0.0199 (3)	−0.0051 (3)
Br3	0.0337 (3)	0.0415 (3)	0.0407 (3)	0.0089 (3)	0.0043 (2)	0.0009 (3)
Br4	0.0450 (4)	0.0397 (3)	0.0446 (4)	0.0155 (3)	0.0205 (3)	0.0109 (3)
Cu1	0.0331 (4)	0.0319 (4)	0.0336 (4)	0.0095 (3)	0.0084 (3)	0.0061 (3)
N1	0.062 (5)	0.074 (5)	0.060 (4)	0.022 (4)	−0.024 (4)	−0.017 (4)
N2	0.034 (3)	0.058 (3)	0.042 (3)	0.002 (3)	0.004 (2)	0.003 (3)
N3	0.053 (4)	0.045 (3)	0.040 (3)	0.002 (3)	0.003 (3)	0.009 (3)
N4	0.050 (3)	0.035 (3)	0.036 (3)	0.005 (2)	0.005 (2)	0.005 (2)
C1	0.078 (6)	0.075 (5)	0.050 (4)	0.052 (5)	0.000 (4)	−0.010 (4)
C2	0.041 (4)	0.077 (5)	0.048 (4)	0.031 (4)	0.014 (3)	0.001 (4)
C3	0.028 (3)	0.046 (3)	0.034 (3)	0.019 (3)	0.005 (2)	0.006 (3)
C4	0.040 (4)	0.060 (4)	0.036 (3)	0.005 (3)	0.010 (3)	0.007 (3)
C5	0.043 (5)	0.083 (6)	0.059 (5)	0.004 (4)	−0.005 (4)	0.014 (5)
C6	0.054 (5)	0.063 (5)	0.074 (5)	−0.005 (4)	0.001 (4)	0.029 (4)
C7	0.058 (5)	0.068 (5)	0.052 (4)	0.009 (4)	−0.003 (4)	−0.011 (4)
C8	0.060 (5)	0.030 (3)	0.048 (4)	0.008 (3)	−0.003 (3)	0.003 (3)
C9	0.050 (4)	0.041 (3)	0.031 (3)	0.014 (3)	0.000 (3)	−0.003 (3)
C10	0.028 (3)	0.036 (3)	0.030 (3)	0.004 (2)	−0.007 (2)	0.001 (3)
C11	0.037 (3)	0.037 (3)	0.034 (3)	0.005 (3)	0.000 (3)	−0.003 (3)
C12	0.038 (4)	0.069 (5)	0.031 (3)	0.015 (3)	0.002 (3)	0.000 (3)
C13	0.060 (5)	0.070 (5)	0.037 (4)	0.009 (4)	0.016 (3)	0.016 (4)
C14	0.091 (6)	0.022 (3)	0.061 (5)	0.007 (3)	0.000 (4)	0.017 (3)

### Geometric parameters (Å, °)

**Table:**

Br1—Cu1	2.4164 (9)	C4—H4	0.9400
Br2—Cu1	2.4039 (9)	C5—H5	0.9400
Br3—Cu1	2.3544 (9)	C6—H6A	0.9700
Br4—Cu1	2.3662 (9)	C6—H6B	0.9700
N1—C5	1.320 (10)	C6—H6C	0.9700
N1—C1	1.330 (11)	C7—H7A	0.9700
N1—H1	0.8800	C7—H7B	0.9700
N2—C3	1.339 (7)	C7—H7C	0.9700
N2—C6	1.458 (9)	C8—C9	1.314 (9)
N2—C7	1.463 (8)	C8—H8	0.9400
N3—C12	1.342 (8)	C9—C10	1.433 (8)
N3—C8	1.343 (9)	C9—H9	0.9400
N3—H3	0.8800	C10—C11	1.410 (8)
N4—C10	1.338 (7)	C11—C12	1.364 (9)
N4—C14	1.457 (7)	C11—H11	0.9400
N4—C13	1.464 (8)	C12—H12	0.9400
C1—C2	1.334 (10)	C13—H13A	0.9700
C1—H1A	0.9400	C13—H13B	0.9700
C2—C3	1.431 (8)	C13—H13C	0.9700
C2—H2	0.9400	C14—H14A	0.9700
C3—C4	1.404 (8)	C14—H14B	0.9700
C4—C5	1.341 (9)	C14—H14C	0.9700
			
Br1—Cu1—Br2	131.05 (4)	N2—C6—H6C	109.5
Br1—Cu1—Br3	99.47 (3)	H6A—C6—H6C	109.5
Br1—Cu1—Br4	97.82 (3)	H6B—C6—H6C	109.5
Br2—Cu1—Br3	100.27 (3)	N2—C7—H7A	109.5
Br2—Cu1—Br4	97.76 (3)	N2—C7—H7B	109.5
Br3—Cu1—Br4	136.48 (4)	H7A—C7—H7B	109.5
C5—N1—C1	119.7 (7)	N2—C7—H7C	109.5
C5—N1—H1	120.2	H7A—C7—H7C	109.5
C1—N1—H1	120.2	H7B—C7—H7C	109.5
C3—N2—C6	122.6 (6)	C9—C8—N3	122.3 (6)
C3—N2—C7	120.4 (6)	C9—C8—H8	118.9
C6—N2—C7	116.7 (6)	N3—C8—H8	118.9
C12—N3—C8	119.5 (6)	C8—C9—C10	120.8 (6)
C12—N3—H3	120.2	C8—C9—H9	119.6
C8—N3—H3	120.2	C10—C9—H9	119.6
C10—N4—C14	122.4 (5)	N4—C10—C11	122.2 (5)
C10—N4—C13	122.4 (5)	N4—C10—C9	121.7 (5)
C14—N4—C13	115.2 (6)	C11—C10—C9	116.1 (6)
C2—C1—N1	122.3 (7)	C12—C11—C10	119.2 (5)
C2—C1—H1A	118.9	C12—C11—H11	120.4
N1—C1—H1A	118.9	C10—C11—H11	120.4
C1—C2—C3	119.6 (7)	N3—C12—C11	122.1 (6)
C1—C2—H2	120.2	N3—C12—H12	118.9
C3—C2—H2	120.2	C11—C12—H12	118.9
N2—C3—C4	123.4 (5)	N4—C13—H13A	109.5
N2—C3—C2	120.7 (6)	N4—C13—H13B	109.5
C4—C3—C2	116.0 (6)	H13A—C13—H13B	109.5
C5—C4—C3	119.5 (6)	N4—C13—H13C	109.5
C5—C4—H4	120.2	H13A—C13—H13C	109.5
C3—C4—H4	120.2	H13B—C13—H13C	109.5
N1—C5—C4	122.9 (8)	N4—C14—H14A	109.5
N1—C5—H5	118.5	N4—C14—H14B	109.5
C4—C5—H5	118.5	H14A—C14—H14B	109.5
N2—C6—H6A	109.5	N4—C14—H14C	109.5
N2—C6—H6B	109.5	H14A—C14—H14C	109.5
H6A—C6—H6B	109.5	H14B—C14—H14C	109.5
			
C5—N1—C1—C2	−0.1 (12)	C12—N3—C8—C9	0.2 (10)
N1—C1—C2—C3	−0.9 (11)	N3—C8—C9—C10	0.5 (10)
C6—N2—C3—C4	176.1 (7)	C14—N4—C10—C11	1.6 (9)
C7—N2—C3—C4	2.7 (9)	C13—N4—C10—C11	−179.0 (5)
C6—N2—C3—C2	−4.6 (9)	C14—N4—C10—C9	−178.1 (6)
C7—N2—C3—C2	−178.0 (6)	C13—N4—C10—C9	1.3 (8)
C1—C2—C3—N2	−178.2 (6)	C8—C9—C10—N4	179.0 (6)
C1—C2—C3—C4	1.2 (9)	C8—C9—C10—C11	−0.7 (8)
N2—C3—C4—C5	178.9 (6)	N4—C10—C11—C12	−179.6 (5)
C2—C3—C4—C5	−0.5 (9)	C9—C10—C11—C12	0.2 (8)
C1—N1—C5—C4	0.8 (12)	C8—N3—C12—C11	−0.8 (9)
C3—C4—C5—N1	−0.4 (11)	C10—C11—C12—N3	0.6 (9)

### Hydrogen-bond geometry (Å, °)

**Table:**

*D*—H···*A*	*D*—H	H···*A*	*D*···*A*	*D*—H···*A*
N1—H1···Br1	0.88	2.54	3.380 (7)	162
N3—H3···Br2	0.88	2.65	3.449 (6)	152
